# Patient safety culture as perceived by operating room professionals: a mixed-methods study

**DOI:** 10.1186/s12913-022-08175-z

**Published:** 2022-06-20

**Authors:** Wiem Aouicha, Mohamed Ayoub Tlili, Jihene Sahli, Ali Mtiraoui, Thouraya Ajmi, Houyem Said Latiri, Souad Chelbi, Mohamed Ben Rejeb, Manel Mallouli

**Affiliations:** 1 Department of Family and Community Health, LR12ES03, University of Sousse, Faculty of Medicine of Sousse, Avenue Mohamed El Karoui, 4002 Sousse, Tunisia; 2 Department of Prevention and Care Safety, Sahloul University Hospital, Route de ceinture Sahloul city 4054, Sousse, Tunisia; 3 Faculty of Medicine of Sousse, Faculty of Medicine, University of Sousse, Avenue Mohamed El Karoui, 4002 Sousse, Tunisia

**Keywords:** Surgical patient safety, Patient safety culture, Operating rooms, Mixed-methods

## Abstract

**Background:**

Routine assessments of patient safety culture within hospitals have been widely recommended to improve patient safety. Experts suggested that mixed-methods studies can help gain a deeper understanding of the concept. However, studies combining quantitative and qualitative approaches exploring patient safety culture are still lacking. This study aimed to explore patient safety culture as perceived by operating room professionals of two university hospitals in Sousse, Tunisia.

**Methods:**

Based on a mixed-methods approach, a cross-sectional survey followed by semi-structured interviews were conducted over a period of two months (December 2019 to January 2020). This study took place in all the operating rooms of two public university hospitals in the district of Sousse, Tunisia. To collect data for this survey, the French version of the Hospital Survey On Patient Safety Culture was used. For interviews, 13 participants were selected purposively using a critical case sampling approach and a topic guide was prepared. Anonymity and confidentiality were respected.

**Results:**

Overall, twelve operating rooms, with different surgical specialties, were included in the study. Survey feedback was provided by 297 professionals representing a response rate of 85.6%. Concerning patient safety culture, the 10 dimensions had low scores (below 50%) and were considered “to be improved”. The highest score was found in ‘teamwork within units’ (45%). Whereas, the lowest scores were allocated to ‘non-punitive response to error’ (22.9%), followed by “frequency of adverse event reported” (25.6%) and “communication openness” (26.3%). Per qualitative data, participants provided a more detailed picture of patient safety issues such as underreporting, absence of an effective reporting system, lack of freedom of expression, and an existing blame culture in operating rooms.

**Conclusions:**

The findings of this study showed a concerning perception held by participants about the lack of a patient safety culture in their operating rooms. It seems essential to design, implement and evaluate strategies that promote a positive patient safety culture and obliterate punitive climate in operating rooms.

**Supplementary Information:**

The online version contains supplementary material available at 10.1186/s12913-022-08175-z.

## Background

For decades, quality of care and patient safety have become major concerns for healthcare facilities [1]. According to the World Health Organization (WHO), patient safety is defined as the “*prevention of errors and adverse events associated with patient care*” [1].

Although there has been a major rise in international interest in patient safety over the past years, Adverse Events (AEs) remain a global challenge and and no country has been able to overcome all patient safety issues [2]. A systematic review, conducted in developed countries by De Vries et al., found that AEs occur in 9.2% of hospital admissions [3].

The situation in developing countries is more critical [4, 5]. For instance, in Africa and the East Mediterranean, estimations show that up to 18% of inpatient admissions were associated with AEs and 3% of hospital admissions were associated with death or permanent disability [6]. Also, another systematic review of 33 studies has reported that healthcare associated infections occur at rates ranging from 5.7 to 19.1% in low- and middle-income countries [5].

A growing body of evidence shows that the majority of in-hospital AEs are associated with surgical care [3, 7]. Consequently, every year, at least seven million patients suffer from surgical complications, including at least one million who die during or immediately after surgery [8].

According to the Higher Authority of Health, the origin of these AEs is rarely related to a lack of technical skills but rather to a lack of safety culture among caregivers [9]. Creating a positive Patient Safety Culture (PSC) was recommended by the Institute of Medicine as an important strategy to improve patient safety and to meet the global challenge posed by AEs [10].

This concept has been defined by the European Society for Quality of Care as: “*a coherent and integrated set of individual and organizational behaviors, based on shared beliefs and values, which continually seeks to reduce related to care harm among patients*. " [11].

A literature review has found a link between safety culture and patient outcomes [12]. For example, an improved PSC resulted in reduced surgical site infections, length of stay and surgical mortality [12–14]. Therefore, PSC measurement has been widely recommended as a routine assessment within hospitals, as it may provide valuable information to organizational leaders regarding strengths and weaknesses in different areas and help design targeted actions to improve patient safety [15].

In most cases, PSC in health care settings is assessed using a questionnaire [16]. Undoubtedly, questionnaires represent a fast and cost-effective approach to collect a maximum of information in a minimum of time and with the least possible effort [16]. However, quantitative data cannot provide in-depth explanations of the obtained scores and limit the possibility to explore the root causes of the detected deficiencies [17, 18].

Hence, to obtain a deeper understanding of the concept, an assessment that combines both quantitative and qualitative methods has been highly recommended [17]. Experts in PSC have suggested that mixed-methods approaches, using interviews or focus groups, can help researchers understand safety culture in a greater depth [17]. Nevertheless, a recently published systematic review, examining methods and tools used to assess PSC in hospitals, highlighted the discrepancy in the large volume of research that exclusively use surveys compared with the considerably lower number of studies combining qualitative and quantitative research [19].

In our study, we sought to expand on the quantitative survey findings and better understand some of the contextual aspects of PSC, particularly in the Operating Rooms (OR) which are a separate unit within hospitals with several specificities that may affect PSC differently from other settings. Besides, and to the best of our knowledge, there isn’t any published study that used a mixed methods approach to assess PSC in this particular setting.

Thus, we aimed to explore PSC as perceived by OR professionals of two large university hospitals in Sousse (Tunisia) using a mixed-methods approach.

## Methods

### Study design and setting

Based on a mixed-methods approach, a cross-sectional survey followed by semi-structured interviews were undertaken, providing a phenomenological context around the data. Data collection was conducted over a period of two months (December 2019 to January 2020). This study took place in all the OR of two public university hospitals in the district of Sousse, Tunisia.

## Stage 1: Questionnaire survey

### Participants

In order to have a representative sample of optimal size, the study was exhaustive and targeted all healthcare professionals (*n* = 347) that were employed in the 12 OR in the aforementioned hospitals, without performing any sampling.

The participants consisted of surgeons, nurses, OR specialized nurses, anesthesiologists, and assistant caregivers. As for the inclusion criteria, they were based on the recommendations of the Hospital Survey On Patient Safety Culture (HSOPSC) questionnaire user’s guide [20].

### Measures

The HSOPSC was originally developed by the Agency for Healthcare Research and Quality (AHRQ) and it is the most extensively used instrument to evaluate PSC [21]. To collect data for this survey, the French version of the HSOPSC provided by the Coordinating Committee for Clinical Evaluation and Quality in Aquitaine in France [22] was used. This French version proposes 10 PSC dimensions and presents favorable psychometric properties; Cronbach’s alpha for the entire questionnaire was 0.88 and ranged from 0.46 to 0.84 for dimensions [22].

Also, reliability was tested for this sample of OR Tunisian caregivers by measuring internal consistency; Cronbach’s alpha was 0.886 for the whole questionnaire and ranged from 0.689 to 0.875 for the 10 dimensions.

Overall, the questionnaire consisted of 51 items: 2 items examined the overall perception of patient safety and the number of AEs reported during the last 12 months, 7 items for demographic and professional characteristics of participants (as seen in Table 1) and 40 items were used to measure ten dimensions related to PSC [20]: (D1) Overall perceptions of patient safety, (D2) Frequency of adverse events reported, (D3) Supervisor/ manager expectations, (D4) Organizational learning – continuous improvement, (D5) Teamwork within units, (D6) Communication openness, (D7) Non-punitive response to errors, (D8) Staffing, (D9) Management support for patient safety and (D10) Teamwork across units.

**Table 1:** Participants’ sociodemographic and professional characteristics

Characteristics		n	%
**Gender**	*Male*	124	41.8
	*Female*	173	58.2
**Professional title**	*Surgeons*	91	30.6
	*Specialized Nurses*	111	37.4
	*Nurses*	91	30.6
	*Assistant caregivers*	04	1.3
**Work Experience**	< *10 years*	159	53.5
	> *10 years*	138	46.5
**OR working years**	< *5 years*	118	39.7
	> *5 years*	179	60.3
**Involvement in risk management committees**	*Yes*	66	22.2
	*No*	231	77.8
**Patient safety training**	*Yes*	148	49.8
	*No*	149	50.2

To rate professionals’ agreement or disagreement, a Likert scale of 5-points was used (from 1 = don't agree at all to 5 = strongly agree), also to estimate frequency (from 1 = never to 5 = always) [22]. If the score of the dimension was 50% or below, it was considered “to be improved” and it reflects a failing PSC. Whereas, if the score was 75% or superior it was considered as “developed” [20]. To have a positive PSC, the 10 dimensions must be developed [20].

### Data collection and ethical considerations

Prior to data collection, the study protocol was reviewed and approved by the institutional ethics committee of the Faculty of Medicine of Sousse and the administrative authorizations from the two hospitals and OR head chiefs were obtained. Then, the investigator handed out a self-reported questionnaire to participants, after explaining the study purposes and outcomes to the participants and obtaining their consent to take part in the study. Also, they were all asked to return the filled in forms to the investigator who immediately placed them in a box. Anonymity and confidentiality were respected.

### Data analysis

The questionnaire consisted of positively and negatively worded items. For items with a positive formulation, “Strongly Agree” and “Agree” or “Most of the time” and “Always” were considered positive responses. For items with a negative formulation, “Strongly Disagree” and “Disagree” or “Never” and “Rarely” were considered positive responses for PSC. Afterwards, for each dimension, a score that represents the average percentage of positive responses to items was calculated. The data analysis was conducted using SPSS version 20 for windows (IBM Corp., Armonk, N.Y., USA), with descriptive analysis displaying the frequencies, percentages, means and standard deviations.

## Stage 2: Semi-structured interviews

### Participants

Following the mixed-methods approach, the second part of the study consisted of semi-structured interviews. Participants were selected purposively from the two included hospitals using a critical case sampling approach in which individuals that are likely to provide the most information are selected [23]. To collect qualitative data, we included at least one participant from each OR until we reached data saturation. First, we randomly listed the 12 ORs, using a random list generator. Then, the recruitment procedure happened with the assistance of the OR supervisors, and the inclusion criterion was a minimum of 3 years of work experience in his current OR (to guarantee a deeper knowledge of the context). Once we had a participant from each unit, we got back to the first OR and the recruitment stopped just after participant 13 because no new relevant information was being obtained (data saturation was achieved).

All the contacted participants (*n* = 13) accepted to take part in the interviews, which were conducted with 4 surgeons (2 seniors and 2 assistants), 4 OR supervisors, 3 OR specialized nurses and 2 anesthesiologists.

### Data collection and ethical consideration

To collect qualitative data, the research team designed an interview topic guide based on prior similar studies [16, 24, 25]. It included 8 questions aiming to assess the main aspects of PSC (Appendix 1).

After elaborating the interview topic guide, it was pilot-tested for lucidity and comprehensibility by giving it to OR professionals, who have all approved of its content. It is important to note that these professionals were excluded from the qualitative data collection.

Prior to data collection, the researchers had first contact with the participants during which they provided information about themselves, their professional background, the study’s objective and why it was important to conduct it. Afterward, to schedule interviews, participants were asked about their availability and time preferences.

The majority of interviews were conducted in the OR supervisor’s office or in a private area in the respective OR without the presence of any other person except the researchers and the interviewee. All the interviews were carried out in the Tunisian/Arabic dialect. As for their duration, it ranged between 26 and 42 min. None of the interviews were repeated.

To collect the data, two researchers (W.A. and MA.T.) were assigned to conduct the interviews. With regard to their background, they were PhD candidates and, originally, OR nurses. Thus, they were familiar with the OR context, which enables them to properly guide the interviews and analyze the data.

After the interviews were audio-recorded, they were transcribed verbatim and the transcripts were reviewed by the participants to make sure that their thoughts were accurately reproduced. Afterward, the final transcripts were translated into English by the main researcher.

Participation in interviews was entirely voluntary and informed consent was given by all interviewees; they were notified that they could refuse or withdraw from the study at any time without any given reason. Besides, before each interview, participants were asked if they minded being audio-recorded and permission to record the interview was granted by all. Also, the collected data were kept anonymous and all identifications were removed before to analysis.

### Data analysis

The research team conducted a thematic analysis of the English transcripts to draw out the significant expressions from each interview. Then, over several meetings, expressions with similar meanings were further studied and subdivided into multiple themes (as shown in Table 4). To minimize the risk of biasing the qualitative assessment by the survey’s findings, the analysis and coding were carried out by two researchers. Notes were compared during several meetings and to resolve any disagreement, we resorted to team consensus.

The findings of the qualitative data were presented to the participants to obtain their feedback and to ensure that their meanings and perspectives were precisely represented.

## Results

### Patient safety culture survey

Overall, twelve operating theatres with different surgical specialties were included in the study. Survey feedback was provided by 297 professionals representing a response rate of 85.6%.

The age of the respondents ranged between 27 and 60 years, with an average of 39.7 ± 9.488 years. Most of the respondents (69.3%, *n* = 206) represented paramedical staff (nurses, specialized nurses, and assistant caregivers) and 31.6% were surgeons. As for work experience, 46.5% (*n* = 138) of participants had a work proficiency of more than 10 years and 60.3% (*n* = 179) worked specifically in OR for more than 6 years (Table 1).

Concerning PSC, the 10 dimensions had low scores (below 50%) and were considered “to be improved”. The highest score was found in D5 ‘teamwork within units’ (45%). Whereas, the lowest scores were allocated to D7 ‘non-punitive response to error’ (22.9%), followed by D2 “frequency of adverse event reported” (25.6%) and D6 “communication openness” (26.3%) (Table 2).

**Table 2:** Scores and items of the 10 dimensions of patient safety culture

Items of patient safety culture dimensions	Absolute frequency (n)	Average positive response (%)
**D1: Overall perceptions of safety**		**33.8**
Patient safety is never sacrificed to get more work done	116	39.2
Our procedures and systems are good at preventing errors from happening	102	34.3
It is just by chance that more serious mistakes do not happen around here	110	37.4
We have patient safety problems in this facility	72	24.2
**D2: Frequency of adverse events reporting**		**25.6**
When a mistake is made, but is caught and corrected before affecting the patient, it is reported	78	26.4
When a mistake is made, but has no potential to harm the patient, it is reported	70	23.6
When a mistake is made that could harm the patient, but does not, it is reported	80	26.9
**D3: Supervisor/manager expectations and actions promoting patient safety**		**36.6**
Manager says a good word when he/she sees a job done according to established patient safety procedures	117	39.7
Manager seriously considers staff suggestions for improving patient safety	100	33.7
Whenever pressure builds up, my manager wants us to work faster, even if it means taking shortcuts	85	28.6
My manager overlooks patient safety problems that happen over and over	129	43.4
**D4: Organizational learning and continuous improvement**		**34**
We are actively doing things to improve patient safety	127	42.8
Mistakes have led to positive changes here	100	33.7
After we make changes to improve patient safety, we evaluate their effectiveness	115	38.7
We are given feedback about changes put into place based on event reports	83	28.1
We are informed about errors that happen in the facility	93	31.4
In this facility, we discuss ways to prevent errors from happening again	87	29.3
**D5: Teamwork within units**		**45**
People support one another in this facility	104	35.3
When a lot of work needs to be done quickly, we work together as a team to get the work done	149	50.2
In facility, people treat each other with respect	141	47.5
When one area in this unit gets really busy, others help out	139	46.8
**D6: Communication openness**		**26.3**
Staff will freely speak up if they see something that may negatively affect patient care	92	31
Staff feel free to question the decisions or actions of those with more authority	52	17.5
Staff are afraid to ask questions when something does not seem right	90	30.3
**D7: Nonpunitive response to error**		**22.9**
Staff feel like their mistakes are held against them	65	22
When an event is reported, it feels like the person is being written up, not the problem	56	18.9
Staff worry that mistakes they make are kept in their personnel file	83	27.9
**D8: Staffing**		**27.2**
We have enough staff to handle the workload	55	18.5
Staff in this facility work longer hours than is best for patient care	77	25.9
We work in crisis mode trying to do too much, too quickly	110	37.2
**D9: Management support for patient safety**		**31.2**
Management provides a work climate that promotes patient safety	92	31.1
The actions of management show that patient safety is a top priority	80	27.1
Management seems interested in patient safety only after an adverse event happens	118	39.7
Units work well together to provide the best care for patients	80	27
**D10: Teamwork across units**		**28.2**
There is good cooperation among units that need to work together	80	26.9
Units do not coordinate well with each other	93	31.3
It is often unpleasant to work with staff from other units	92	31
Things ‘fall between the cracks’ when transferring patients from one unit to another	94	31.6
Important patient care information is often lost during shift changes	79	26.6
Problems often occur in the exchange of information across units	64	21.5

The level of patient safety in the OR was deemed ‘Acceptable' in 55.6% of cases and ‘Poor’ in 26.3%. Regarding reported AEs, 85.9% of the participants declared that they did not report any event in the last 12 months (Table 3).

**Table 3:** The level of patient safety perceived and the number of reported AEs during the last 12 months

**Level of perceived patient safety**	**n**	**%**
* Excellent*	07	2.4
* Very good*	24	8
* Acceptable*	165	55.6
* Poor*	78	26.3
* Failing*	23	7.7
**Number of adverse events reported in the past 12 months**	**n**	**%**
* No event reported*	255	85.9
* 1–2*	20	6.7
* 3–5*	13	4.4
* 6–10*	08	2.7
* 11–20*	1	0.3

### Interviews results

The age of the interviewees ranged from 32 to 47 years and most of them were women (8 out of 13). The majority of them had more than 10 years of work experience (10/13) and more than 12 working years in their OR (7/13).

Thematic analysis revealed six main themes (i.e., patient safety concerns, failing AEs reporting, teamwork, poor working conditions, and hospital management obstacles) that were extracted from the 13 interviews (Table 4).

**Table 4:** Factors influencing patient safety culture in the OR: themes, subthemes and main codes

Themes	Subthemes	Main codes
**Patient safety concerns**	Theory–practice gap	lack of compliance to guidelines, existing knowledge (trainings, educational flyers and posters…), struggle to integrate knowledge into practice,
	Misbehavior	Ranging from laziness, lack of dynamism, tardiness and absenteeism, delaying the surgical schedule, cancelling scheduled surgeries
	Equipment failures	Missing or unfunctional equipment leading to surgical schedule delay, an improper or lack of scheduled maintenance of OR equipment,
**Teamwork**	Sufficient support	Sufficient respect & mutual support
	Communication issues	Lack of communication, absence of communication openness, divided team, tension impairing communication, nurse/physician relationship, difficulties concerning information sharing and inclusion in the decision-making process
**Failing AEs reporting**	Underreporting	Not everything is reported, absence of an effective incident reporting system, lack of reactivity,
	Lack of freedom of expression	Professionals don’t feel free to talk about errors or report them
	Blame culture	perceived culpability and fear of punishment when reporting an error, committing errors is treated as a lack of skills or recklessness
**Poor working conditions**	Staff shortages	Inadequate staffing compromising patient safety, unsatisfying working conditions, shifting towards the private sector,
	High workload	excessive workload, a constant climate of pressure and stress, error inducing
**Hospital management obstacles**	Inappropriate risk management strategies	Necessity of proactive strategies, should predict system weaknesses to minimize patient harm
	Absence of adequate supervision	insufficient supervision leading to disrespect of protocols, the importance of a constant practice evaluation
	Lack of training opportunities	Existing barriers to training adherence, difficulty to have permissions, lack of scheduled educational sessions, discrepancy between teams in terms of facilitating trainings

### Patient safety concerns

Most of the interviewees (8/13) declared that the care provided in their OR was relatively safe, but that it could be improved. All the participants felt that there were many issues that threaten patient safety on a daily basis.

### Theory–practice gap

The majority of the interviewees (10/13) highlighted that practitioners often struggle to integrate knowledge learned in training sessions with real-world OR practice.

“Most nurses and technicians in my unit know the importance of the surgical site preparation and how it is supposed to be done, however, it is not always performed and if it is, usually it’s sloppy work that does not respect the guidelines”. Nurse 2.

“Despite the display of many educational posters in the OR serving as reminders to ensure patient safety, I feel that compliance with these guidelines remains low. For instance, in my OR, the surgical hand scrub, which is a process with determined steps and duration, is rarely correctly performed”. Nurse 6.

### Misbehavior

Participants pointed out that acquiring knowledge and attending training sessions do not necessarily lead to a good surgical practice due to several reasons such as misbehavior of OR workers (5/13). According to the interviews, the aspects of misbehavior ranged from laziness or lack of dynamism to tardiness and absenteeism. Most participants (7/13) recalled cases of tardiness and absenteeism that happened in their OR and stressed that it posed a threat to patient safety and the working climate.

“I remember one day; we had an urgent surgery first thing in the morning and the anesthesiologist was late. He couldn’t be replaced at the moment, which led to canceling the surgery that day”. Surgeon 1.

“Sometimes, in between surgeries, the cleaning worker is nowhere to be found and there is no one to clean the OR and prepare it for the next surgery. This neglect usually results in delaying the surgical schedule or canceling scheduled surgeries”. Nurse 5.

### Equipment failures

Equipment failures were highlighted by the majority of interviewees (11/13) as the most common cause of surgical schedule delays in their OR.

“Equipment failures are frequent in OR; it’s common that surgeries get delayed just because either the equipment is damaged or we don’t find the needed one. No doubt, you heard about the surgical light head that broke down during a C-section and the surgical team had to carry out the rest of the intervention using phones flashlight”. Surgeon 2.

“Sometimes, the operation has to stop momentarily until we find another electric scalpel”. Anesthesiologist 1.

According to participants, equipment failures, in most cases, were caused by maintenance omissions, such as an improper or lack of scheduled maintenance of OR equipment.

“We only see the maintenance team, when something is broken down. It shouldn’t be the case; maintenance of OR equipment should be performed periodically and appropriately”. Surgeon 2.

### Teamwork

#### Sufficient support

Regarding teamwork, participants believed that it was fairly acceptable in their OR. More than half of them felt that there was sufficient mutual support between them and their fellows (7/13).

“You spend more than half of your awake time in the OR, surrounded by your colleagues. If you don’t feel supported and respected, I am not sure you can do your job correctly”. Nurse 5.

### Communication issues

The majority highlighted the importance of effective communication to ensure a fluid surgical workflow in the OR (11/13). However, during interviews, participants expressed dissatisfaction with the quality of communication in their unit (9/13).

“It feels like we are many different teams: nurses, surgeons, anesthesiologists, and each team works solely. For instance, we are never included in briefings/debriefings as a whole multidisciplinary team”. Anesthesiologist 1.

Also, participants stressed that facing difficulties during surgery often results in creating tension that impairs teamwork and weakens communication (4/13).

“When it gets tense during an operation, the senior surgeon gets pissed off, assumes that it’s his responsibility only to save the patient, and starts ordering everyone around, sometimes, even in a disrespectful manner.” Nurse 1.

In terms of nurse-physician relationship, nurses made clear that there were some difficulties concerning information sharing and participation in the decision-making process.

“Not all information is shared among the team. As a nurse, it feels like we are omitted from discussions around patient care”. Nurse 1.

“I don’t think that we understand teamwork correctly. According to some, nurses should not be included in the decision-making process. Their sole role is to take orders from surgeons and execute them”. Nurse 3.

### Failing AEs reporting

#### Underreporting

During interviews, participants asserted that not everything is reported in the OR (12/13).

“Many near-misses and errors happen and they are not reported just because the problem is usually resolved without any harm to the patient. This leads to the recurrence of such events because we did not treat their root causes.” Surgeon 3.

In addition, participants noted the absence of an effective incident reporting system. They stressed that motivated personnel usually have to make an extra effort to report AEs and see them through to the analyzing committee, but the existing reporting system lacks reactivity and it doesn’t go any further from there.

“Look, in my unit, personnel involved in patient safety training and motivated to ensure a better quality of care do report AEs but, in most cases, there isn’t a response and it doesn’t go any further, which I find it, personally, frustrating”. Anesthesiologist 2.

### Lack of freedom of expression

With regard to communication openness, participants declared that they don’t feel free to talk about errors or report them (5/13). This issue wasn’t only associated with supervisors but also with fellow colleagues.

“I feel like I don’t have the liberty to report AEs, and if I do, I will be treated as a snitch and get called out for turning my back to my colleagues. It happened to me before, and it wasn’t a good place. I mean I tried to explain that it wasn’t personal, that it was the right thing to do… but they didn’t listen”. Nurse 4.

### Blame culture

All the participants associated the underreporting in their OR with a perceived culpability and a fear of punishment when they needed to disclose an error.

“Committing errors is treated generally as a lack of skills and recklessness. The person who commits an error is accused and it is rarely considered as a shared responsibility of the team”. Surgeon 1.

### Poor working conditions

#### Staff shortages

All the interviewees thought that there wasn’t enough personnel and that the lack of staff in their OR compromised patient safety. Particularly, according to most participants (9/13), the unsatisfying working conditions have led to a strong shift towards the private sector and reinforced the staff shortages in hospitals.

“Due to inadequate working conditions, so many “good” surgeons are leaving the hospital and migrating to the private sector without being replaced”. Surgeon 3.

### High workload

Regarding workload, it was noted that most participants (8/13) weren’t satisfied with how much work they handled during their daily shifts. Some acknowledged that sometimes it gets chaotic in the OR and they fail to manage the excessive workload (3/13). Also, they stressed that the latter created a climate of pressure and stress, and was error inducing.

“On busy days, which are common, I wish I could press hold and take a breath. My only concern is that if I am tired, I won’t do my job right and the patient could suffer”. Nurse 7.

“The orthopedic surgical unit is the only unit of its kind in all of central Tunisia so it gathers thousands of patients from so many districts, resulting in a huge workload that we have to handle with limited staff. It is tiring!” Surgeon 3.

### Hospital management obstacles

Most informants highlighted the importance of hospital management to ensure surgical patient safety (7/13). They recognized all the efforts made by the management, pointed out the weaknesses and suggested some areas of improvement.

### Inappropriate risk management strategies

The interviewed OR staff (5/13) stressed that the hospital should focus more on risk management and reinforce proactive strategies that identify and predict system weaknesses and adopt changes to minimize patient harm.

“It seems to me that the hospital management moves only after an accident happens.” Surgeon 3.

“As an OR supervisor, I know that double gloving is recommended to lower the risk of surgical site infections and pathogen transfer, yet when I ask for more gloves, the hospital management asks me to reduce glove usage. Don’t they know that double gloving costs less than surgical site infections?”. Nurse 6.

### Absence of adequate supervision

The majority of participants (10/13) made clear that adherence to existing protocols and guidelines was not constantly observed, and the main reason was that there wasn’t sufficient supervision.

“No matter how many printed protocols and guidelines are displayed, their application remains limited because there is a huge lack of supervision”. Nurse 2

More than half of the interviewees (8/13) felt the importance of a constant practice evaluation aiming to increase protocols respect and prevent AEs and errors in the OR.

“I believe that if people knew that they could be supervised at any moment, it would definitely improve their respect and compliance with the guidelines”. Nurse 7.

### Lack of training opportunities

During interviews, participants stressed that training and continuous education were essential to keep up with surgical practice progress. They pointed out that the quality of trainings they were receiving was fairly satisfying (7/13). However, some issues were raised as barriers to training adherence, such as the difficulty to have permission and the lack of scheduled educational sessions.

“Usually, if there is a training session, you would have to obtain the OR supervisor’s permission and find someone to replace you. It’s like you are going on a vacation but you are not!!”. Nurse 1.

“Sometimes we learn about new techniques and information from junior staff and trainees… It is embarrassing! Aren’t we supposed to have scheduled training sessions to update our knowledge?”. Nurse 4.

Besides, three nurses, during interviews, declared that the medical staff benefits from a more solid training program and is privileged by the hospital management in terms of granting leaves and funds.

## Discussion

Recently, routine assessments of PSC within hospitals have been widely recommended to improve patient safety [15]. The survey results indicated that all the PSC dimensions needed improvements (scores below 50%). In fact, these findings reflect an alarming situation in the OR and a failing system in terms of patient safety. This could be explained by the professionals’ lack of information and awareness regarding the different domains of PSC.

### Overall perception of safety

According to our findings, only 33.8% of participants had a positive overall perception of safety (D1) in their OR. According to Verbeek-van Noord et al., the perceptions of caregivers regarding patient safety in their units could be relevant and revealing, as they might be the first to notice safety issues [26]**.** In fact, 75.8% of the study participants declared that they have patient safety problems in this facility, and only 34.3% stated that the existing procedures are efficient to prevent errors from happening. These results were confirmed during interviews as all the interviewees felt that there was an array of factors that jeopardize patient safety, such as theory–practice gap, misbehavior, and equipment failures. With regard to equipment failures, Wubben et al. found that equipment-related incidents occurred frequently in the OR (up to 15.9%) and resulted in an extra work and additional minutes of delay per event [27]**.** In fact, obtaining functioning medical equipment is a challenge particularly in low- and middle-income countries [28]. According to WHO estimations, 50%-80% of medical equipment in developing countries is either deficient or failing as it lacks assessment systems and regular maintenance, which prevents the delivery of adequate care to patients [28].

### AEs reporting

Survey data revealed that ‘non-punitive response to error’ had the lowest score. Findings from other studies conducted in the OR found that this dimension also had a very low score [25, 29]**.**

Qualitative data supported survey results and provided a more detailed picture of patient safety issues such as underreporting, absence of an effective reporting system, lack of freedom of expression, and an existing blame culture. During interviews, participants pointed out that errors were recurrent because usually, in case of an error, immediate corrective actions were taken without addressing the root causes. In fact, according to Argyris, reporting errors is considered as double-loop learning, starting by detecting errors, learning from them and proactively treating the underlying causes to prevent their reoccurrence [30].

Along with that, results have shown that 85.9% of the respondents haven’t reported any AEs in the past 12 months, proving the absence of an AEs reporting system, which was addressed by the majority of interviewees. In fact, an effective error reporting system is crucial to establish a reporting culture in which people feel safe pointing out and reporting errors without fear of retaliation [31].

However, there is an existing punitive climate that was corroborated by the qualitative and quantitative data. For the most part, fear of discoverability, reprisals, and possible litigation are still real challenges making error reporting uncommon and menacing for surgical patient safety [32].

### Hospital management and lack of supervision

With regard to hospital management, the absence of adequate supervision was pointed out as the reason for non-adherence to existing protocols and guidelines. Studies by Labat et al. [33] and Abdi et al. [24] have raised the same statement as they found that evaluation of practice aiming to prevent AEs was not implemented, leading to poor compliance with evidence-based guidelines by care providers.

Clinical supervision is a professional activity where the more experienced clinician supervises the knowledge and experience of the less experienced ones [34]. This supervision allows to address any gaps in knowledge or skill set, thereby improve the clinical performance and quality of care [35]. For instance, in a systematic review that investigated the impact of clinical supervision, Snowdon et al. concluded that clinical supervision is associated with safer surgery, such as reduced mortality and complications [36].

### Recommendations for practice

In light of our findings, we recommend the standardization of the Surgical Safety Checklist (SSC) in the Tunisian context. Using the SSC can prevent communication and equipment failures and decreases the likelihood of committing errors, especially in a distraction-prone environment like the OR [37, 38]. Also, to improve communication and teamwork, we recommend preoperative briefings that include not only surgeons but also nurses and anesthesiologists. These have been shown to be positively associated with better patient safety attitudes and better teamwork [39].

In addition, and to help bridge the gap between theory and practice, we recommend the use of clinical simulation. Indeed, there is a growing body of research that supports the idea that simulated experiences can offer an engaging teaching method to reinforce and evaluate competence in practical skills, critical thinking and decision-making through the use of knowledge, promoting a connection between theory and practice [40, 41].

Regarding AEs, an effective incident reporting system should be installed that includes error identification, reporting, analysis and corrective actions. Importantly, reporting errors should be encouraged and endorsed by supervisors as an integral part of a continuous cycle of improving patient safety, and quality of care and confidentiality and legal protection are therefore required to encourage health practitioners to come forward [42].

### Strengths and limitations

To the best of our knowledge, this is the only mixed-methods study that focused on PSC in OR. In fact, experts suggested that mixed-methods studies can help gain a deeper understanding of the concept. Furthermore, studies combining quantitative and qualitative approaches to explore PSC are still lacking in the literature [17].

As for the study limitations, it was conducted in two public hospitals in Sousse (Tunisia) which may limit the generalizability of the findings to other healthcare settings and geographical areas. For future studies, it is suggested to perform a nationwide study including more Tunisian hospitals.

Another limitation was related to a self-reporting bias which is common in this type of studies. In fact, when answering a self-reported questionnaire, participants may fear of being identified and, thus, may change or distort their responses. To reduce this bias, the investigator emphasized the confidential and anonymous nature of the study (all the filled forms were placed in a box).

Additionally, qualitative data are, also, prone to a reporting bias as participants may not give answers that are fully correct, either because they do not know the full answer or because they seek to make a good impression. Stopping interviews only after reaching data saturation can limit the impact of this bias. In future studies, we suggest that even after reaching data saturation, further interviews should take place (with one or two more participants) to ensure that data saturation was really reached, which is a common practice in qualitative studies [43].

## Conclusions

The findings of this study showed a concerning perception held by participants about the lack of a PSC in their OR. Indeed, the 10 dimensions had very low scores and were all in need of improvement. We highlighted different areas of concern, such as frequency of AEs reported, non-punitive response to error, teamwork, and staffing. Per qualitative data, participants provided a more detailed picture of patient safety issues such as underreporting, absence of an effective reporting system, lack of freedom of expression and an existing blame culture in OR.

For future studies, it is suggested to implement the recommended strategies and evaluate their effectiveness to improve patient safety attitudes and practices in the OR.

## Supplementary Information

**Additional file 1: Appendix 1. **Interview topic guide.

## Data Availability

The datasets used and/or analyzed during the current study are available from the corresponding author on reasonable request.

## Abbreviations

PSC: Patient Safety Culture
AEs: Adverse Events
OR: Operating Rooms
WHO: World Health Organization
HSOPSC: Hospital Survey On Patient Safety Culture
AHRQ: Agency for Healthcare Research and Quality
SSC: Surgical Safety Checklist
