# Supplementary material for: Patient safety culture as perceived by operating room professionals: a mixed-methods study
Source: BMC Health Serv Res. 2022 Jun 20;22:799. doi: 10.1186/s12913-022-08175-z (PMC9210674; doi:10.1186/s12913-022-08175-z)
Supplement: Supplementary file 1 — Additional file 1: Appendix 1. Interview topic guide. [file 12913_2022_8175_MOESM1_ESM.docx]

**Interview topic guide**

**Q1:** How safe do you think care is in your OR?

**Q2:** How often AEs occur in your OR? And what do you believe are the causes?

**Q3:** If you notice that something care-related is wrong, do you feel free to share your concerns? Why?

**Q4**: In your OR, how people who report errors are treated? And why?

**Q5:** What do you think of teamwork in your OR?

**Q6:** What do you think about the workload in your OR?

**Q7:** What do you think about hospital management?

**Q8:** What do you suggest to improve patient safety in your OR?
